# Comparison of the effects of pemafibrate and omega-3 fatty acid ethyl on fatty liver index in patients with dyslipidemia treated with statin: a sub-analysis from the PROUD48 study

**DOI:** 10.3389/fendo.2025.1549687

**Published:** 2025-05-01

**Authors:** Yasutaka Takeda, Masato Furuhashi, Ichiro Sakuma, Shinya Hiramitsu, Mizuho Okada, Shinichiro Ueda, Naoki Kumashiro, Masaru Sakurai

**Affiliations:** ^1^ Department of Diabetology & Endocrinology, Kanazawa Medical University, Uchinada, Japan; ^2^ Department of Cardiovascular, Renal and Metabolic Medicine, Sapporo Medical University School of Medicine, Sapporo, Japan; ^3^ Caress Sapporo Hokko Memorial Clinic, Sapporo, Japan; ^4^ Hiramitsu Heart Clinic, Nagoya, Japan; ^5^ Keiyukai Yoshida Hospital, Asahikawa, Japan; ^6^ Department of Clinical Pharmacology and Therapeutics, University of the Ryukyus, Nishihara, Japan; ^7^ Department of Social and Environmental Medicine, Kanazawa Medical University, Uchinada, Japan

**Keywords:** fatty liver index, atherosclerotic cardiovascular disease, residual risk, hypertriglyceridemia, nonalcoholic fatty liver disease, metabolic dysfunction-associated fatty liver disease, metabolic dysfunction-associated steatotic liver disease

## Abstract

**Background:**

Fatty liver index (FLI) calculated by using body mass index, waist circumference and levels of triglycerides and γ-glutamyl transpeptidase is a noninvasive biomarker for diagnosis of metabolic dysfunction-associated steatotic liver disease (MASLD), which is one of the high-risk conditions of atherosclerotic cardiovascular diseases. To compare the effects of pemafibrate and omega-3 fatty acid ethyl on FLI, we conducted a sub-analysis study of the Pemafibrate Reduction of triglyceride-rich lipoproteins compared with Omega-3 fatty acid ethyl for Unmet needs in Dyslipidemic patients on target to apoB-48 (PROUD48) study.

**Methods:**

57 participants in the pemafibrate 0.4 mg per day treatment group (PEMA, men/women: 37/20, mean 64 years) and 60 participants in the omega-3 fatty acid ethyl 4 g per day treatment group (OMEGA-3, men/women: 35/25, mean 63 years) in the PROUD48 study were included in the present study. Changes in FLI and prevalence of MASLD from baseline to week 16 in PEMA and OMEGA-3 were investigated.

**Results:**

Median FLI was significantly decreased by both PEMA (69.7 to 47.6, *P* < 0.001) and OMEGA-3 (64.8 to 59.5, *P* < 0.001). There was a significant difference in change in FLI between PEMA and OMEGA-3 (-18.3 ± 14.1 vs. -5.5 ± 9.4, *P* < 0.001). The proportions of MASLD estimated by FLI (baseline/week 16) in PEMA and OMEGA-3 were 93.0/68.4% (*P* = 0.002) and 90.0/85.0% (*P* = 0.582), respectively.

**Conclusions:**

Pemafibrate is superior to omega-3 fatty acid ethyl in lowering effects of FLI and MASLD in patients with dyslipidemia receiving statin treatment, suggesting that pemafibrate is a beneficial agent for hypertriglyceridemia and reduction of the risk for MASLD.

## 1 Introduction

To prevent atherosclerotic cardiovascular disease (ASCVD), there is no doubt that low-density lipoprotein cholesterol (LDL-C)-lowering therapy is of paramount importance. A reduction of 1 mmol/L in LDL-C level by treatment with statins has been shown to reduce the incidence of major vascular events by 25% in individuals without prior ASCVD (1). However, the remaining > 70% incidence rate is known as the residual ASCVD risk, which includes a high level of triglycerides (TG) and a low level of high-density lipoprotein cholesterol (HDL-C) (2, 3).

Recently, we prospectively compared the lowering effects of pemafibrate, a selective peroxisome proliferator-activated receptor α (PPARα) modulator, and omega-3 fatty acid ethyl, polyunsaturated fatty acids, on levels of fasting apolipoprotein B-48 (apoB-48), a surrogate marker that reflects postprandial hypertriglyceridemia, in the Pemafibrate Reduction of triglyceride-rich lipoproteins compared with Omega-3 fatty acid ethyl for Unmet needs in Dyslipidemic patients on target to apoB-48 (PROUD48) study (4). The PROUD48 study demonstrated that pemafibrate was superior to omega-3 fatty acid ethyl in a lowering effect of fasting apoB-48 and also provided a new clinical insight that pemafibrate was a better option for pharmacotherapy of hypertriglyceridemia to reduce the residual ASCVD risk in patients with dyslipidemia receiving statin treatment (5).

Nonalcoholic fatty liver disease (NAFLD) is the most common chronic liver disease, in which global prevalence is approximately 25% and its incidence has been increasing (6). Some patients with simple steatosis in NAFLD progress to a more severe form nonalcoholic steatohepatitis (NASH) and develop liver cirrhosis and hepatocellular carcinoma. In recent years, NAFLD has also been considered to be a high-risk condition for ASCVD (7, 8).

An international panel of experts recently proposed metabolic dysfunction-associated fatty liver disease (MAFLD) independent of alcohol consumption as a new concept for fatty liver to replace NAFLD (9). The criteria for MAFLD are based on evidence of hepatic steatosis in addition to one of the following criteria: overweight/obesity, type 2 diabetes, and evidence of metabolic dysregulation. Subsequently, in 2023, three large multinational liver associations proposed to replace the term NAFLD with metabolic dysfunction-associated steatotic liver disease (MASLD) (10). They also proposed to choose metabolic dysfunction-associated steatohepatitis (MASH) as an alternative name to NASH. Furthermore, previous studies demonstrated that 95~99% of patients with NAFLD met the criteria of MASLD (11, 12).

Although the gold standard for diagnosis of NAFLD/MASLD is liver biopsy, several noninvasive diagnostic markers for NAFLD/MASLD have been established (9, 13). Fatty liver index (FLI), a biomarker for detection of hepatic steatosis proposed by Bedogni et al. in 2006, is calculated by using body mass index (BMI), waist circumference (WC) and levels of TG and γ-glutamyl transpeptidase (γ-GTP) (14). Previous studies showed that FLI closely corresponds to findings of histology and imaging modalities of NAFLD/MASLD (12, 15–17).

A previous study conducted in Japan showed that treatment with pemafibrate significantly improved levels of alanine aminotransferase (ALT), γ-GTP and alkaline phosphatase (ALP) in patients with NAFLD compared with placebo (18). Interestingly, the PROUD48 study also revealed that pemafibrate was superior to omega-3 fatty acid ethyl in lowering levels of ALT, γ-GTP, and ALP (5). Considering the relationship between MASLD and ASCVD risk, the favorable effect of pemafibrate on liver function is expected to have an additional impact on the reduction of ASCVD risk. However, the effects on MASLD have not been verified yet. To elucidate the effects of pemafibrate and omega-3 fatty acid ethyl on MASLD, change in FLI and the prevalence of MASLD based on the detection of hepatosteatosis by FLI were investigated in dyslipidemic patients with statin treatment as a sub-analysis of the PROUD48 study.

## 2 Methods

### 2.1 Study design

This study is a sub-analysis of the PROUD48 study, which was a prospective, multicenter, open-label, randomized, parallel group, comparative trial to compare the effects of pemafibrate and omega-3 fatty acid ethyl on the fasting apoB-48 level, a surrogate marker for postprandial hypertriglyceridemia in patients with dyslipidemia. The detailed rationale, design, and protocol of the PROUD48 study were previously described (4, 5). The PROUD48 study was registered in the Japan Registry of Clinical Trials (jRCT) on the 28^th^ of April 2020 (No. jRCTs071200011).

### 2.2 Study participants

Participants were recruited from the PROUD48 study. The PROUD48 study was conducted in Japanese patients with dyslipidemia in accordance with the principles of the Declaration of Helsinki and its amendments. Participants in the PROUD48 study were ambulatory patients who presented to Asahikawa Medical University Hospital (Asahikawa, Hokkaido), Caress Sapporo Hokko Memorial Clinic (Sapporo, Hokkaido), Hiramitsu Heart Clinic (Nagoya, Aichi, Japan) and Keiyukai Yoshida Hospital (Asahikawa, Hokkaido). The inclusion criteria were ambulatory patients with dyslipidemia receiving statin treatment for more than 4 weeks, with fasting TG levels of ≥ 177 mg/dL (2 mmol/L); aged 20–79 years; and those who provided written informed consent. The exclusion criteria were fasting TG levels ≥ 500 mg/dL (5.7 mmol/L); diabetic patients with HbA1c levels ≥ 9% and who need insulin treatment; type 1 diabetes; serum creatinine levels ≥ 1.5 mg/dL or higher; patients who used fibrates and nicotinic acids within 4 weeks; patients who used polyunsaturated fatty acids including supplements within 24 weeks; symptomatic cardiovascular and cerebrovascular disorders; severe infections; acute hepatitis or liver cirrhosis; cancer; patients before or after surgery; women with pregnancy or during breastfeeding; patients who need lipid management with proprotein convertase subtilisin/kexin type 9 inhibitors or microsomal triglyceride transfer protein inhibitors; patients who have contraindications for pemafibrate and omega-3 fatty acid ethyl (4, 5). Written informed consent for participation was obtained from all participants prior to randomization. The study protocol was approved by the Certified Review Board of the University of the Ryukyus for Clinical Research Ethics (No. CRB7200001). Among 129 participants who were enrolled in the PROUD48 study, a total of 117 participants were eventually included in the present study.

### 2.3 Randomization and intervention

The randomization and intervention were also described in our previous reports (4, 5). The participants were randomly allocated to the pemafibrate treatment group (PEMA) or omega-3 fatty acid ethyl treatment group (OMEGA-3) in a 1:1 ratio. Participants in the PEMA were given pemafibrate at a dose of 0.2 mg orally twice a day for 16 weeks with continuing statin treatment. Participants in the OMEGA-3 were given omega-3 fatty acid ethyl at a dose of 2 g orally twice a day for 16 weeks with continuing statin treatment. During the study, the addition of new drugs, discontinuation, or dose changes of all drugs including statins, pemafibrate, and omega-3 fatty acid ethyl were not permitted. Based on the Japan Atherosclerosis Society guidelines, all participants were on a diet with an optimized total energy intake based on their ideal body weight and daily activity to maintain an appropriate body weight during the study (19).

### 2.4 Measurements

To profile the study population, clinical characteristics potentially associated with MASLD including age, sex, comorbidities, habits, WC, BMI, lipids-related parameters (total cholesterol, TG, LDL-C, and HDL-C), glycemic control-related parameters (fasting plasma glucose, fasting immunoreactive insulin [IRI], homeostasis model assessment insulin resistance [HOMA-IR], and HbA1c), and other blood biochemical parameters (aspartate aminotransferase [AST], ALT, γ-GTP, ALP, creatinine, and estimated glomerular filtration rate [eGFR]) were extracted and reiterated. Levels of total cholesterol, TG, LDL-C, HDL-C, IRI, and HbA1c were measured at a central clinical laboratory (SRL, Hachioji, Japan). Other clinical parameters were measured at each institution. HOMA-IR was calculated based on a previous report (20), and eGFR was calculated with serum creatinine, sex, and age using the established equation for Japanese subjects (21).

FLI was calculated by using BMI, WC and levels of TG and γ-GTP (14). Although the cutoff value for steatotic liver disease (SLD) was originally reported as FLI ≥ 60 in Italian subjects, FLI ≥ 35 for Japanese men and FLI ≥ 16 for Japanese women were used for the detection of SLD for diagnosis of MASLD as previously reported (12, 17).

MASLD was diagnosed by the absence of other discernible causes for hepatic steatosis and the presence of SLD with at least one of five cardiometabolic risk factors including 1) BMI ≥ 23 kg/m^2^ or WC > 90/80 cm in Asian men and women; 2) fasting glucose ≥ 100 mg/dL, 2-h post-load glucose levels ≥ 140 mg/dL (no measurement in the present study), HbA1c ≥ 5.7%, type 2 diabetes mellitus, or treatment for type 2 diabetes mellitus; 3) blood pressure ≥ 130/85 mmHg or specific antihypertensive drug treatment; 4) plasma TG ≥ 150 mg/dL or lipid-lowering treatment; and 5) plasma HDL-C ≤ 40 mg/dL for men and ≤ 50 mg/dL for women or lipid-lowering treatment (10). The category of MASLD and increased alcohol intake (MetALD) diagnosed by the presence of MASLD and average alcohol intake of 140–350 g/week (20–50 g/day) for women and 210–420 g/week (30–60 g/day) for men was also included (10).

### 2.5 Statistical analysis

Continuous and categorical variables were presented as means ± standard deviations (SDs), medians (interquartile ranges [IQR] or min-max values [Min-Max]) and frequencies with percentages. The Shapiro-Wilk test was used to assess the normality of data. Changes in FLI from baseline to week 16 were compared by using unpaired *t* test. The others were analyzed using the paired *t* test or Wilcoxon’s signed-rank test for intragroup comparisons and the unpaired *t* test or Mann-Whitney *U* test for comparisons between two groups. The baseline characteristics of the participants in the two groups were compared using the chi-square test or Fisher’s exact test for categorical variables and *t* test or Mann-Whitney *U* test for continuous variables. All *P*-values were two-sided with *P* < 0.05 taken to indicate statistical significance. All statistical analyses were performed by the study statistician (M. Sakurai) at the data center (Nexis, Fukuoka, Japan) using SPSS ver. 26 (IBM Corp., Armonk, NY, USA).

## 3 Results

### 3.1 Participants and baseline characteristics

As previously shown in the PROUD48 study (5), a total of 129 participants were recruited and assessed for eligibility. Three participants were excluded because of withdrawal of consent and failure to visit. The remaining 126 participants were randomly assigned to the PEMA group (n = 63) and the OMEGA-3 group (n = 63). After exclusion of patients who discontinued the treatment, 58 participants in the PEMA group and 61 participants in the OMEGA-3 were followed-up. Eventually, 57 participants (men/women: 37/20, mean 64 years) in the PEMA group and 60 participants (men/women: 35/25, mean 63 years) in the OMEGA-3 group completed the follow-up by May 22, 2021 (5) and were included in the present study.

Baseline characteristics were well balanced between the two groups except for the prevalence of hypertension and levels of TG and ALP (**Table 1**). The median levels of FLI in the PEMA group and the OMEGA-3 group at baseline were 69.7 and 64.8, respectively.

**Table 1:** Baseline characteristics of participants.

	All				PEMA				OMEGA-3				*P*
	n				n				n				
Age (years)	117	63.5	±	9.7	57	64.4	±	8.6	60	62.7	±	10.6	0.331
Sex, women, n (%)	117	44	(37.6)		57	19	(33.3)		60	25	(41.7)		0.352
Diabetes mellitus, n (%)	117	55	(47.0)		57	25	(43.9)		60	30	(50.0)		0.506
Hypertension, n (%)	117	82	(70.1)		57	46	(80.7)		60	36	(60.0)		0.015
Habitual alcohol intake_yes, n (%)	117	73	(62.4)		57	34	(59.6)		60	39	(65.0)		0.550
Current-smoker, n (%)	117	29	(24.8)		57	13	(22.8)		60	16	(26.7)		
Waist circumference (cm)	116	93.3	±	9.6	56	94.1	±	8.9	60	92.5	±	10.3	0.380
BMI (kg/m^2^)	117	26.4	±	4.1	57	26.6	±	3.4	60	26.2	±	4.6	0.583
Total cholesterol (mg/dL)	117	181.9	±	25.4	57	182.0	±	23.8	60	181.7	±	27.1	0.946
TG (mg/dL)	117	207	(71–492)		57	198	(76–380)		60	220	(71–492)		0.042
HDL-C (mg/dL)	117	49.5	±	11.5	57	50.2	±	12.2	60	48.9	±	10.9	0.531
LDL-C (mg/dL)	117	97.0	±	23.4	57	97.5	±	24.5	60	96.4	±	22.6	0.790
Fasting plasma glucose (mg/dL)	117	118.8	±	27.0	57	121.0	±	31.8	60	116.8	±	21.6	0.410
HbA1c (%)	117	6.2	±	0.9	57	6.2	±	1.0	60	6.1	±	0.8	0.682
IRI (IU/L)	117	9.02	(1.63–110)		57	9.25	(2.59–110)		60	8.96	(1.63–32.0)		0.681
HOMA-IR	117	2.54	(0.39–39.4)		57	2.35	(0.74–39.4)		60	2.61	(0.39–7.38)		0.689
AST (IU/L)	117	25	(13–110)		57	25	(13–110)		60	25	(13–97)		0.396
ALT (IU/L)	117	28	(10–174)		57	29	(13–162)		60	26	(10–174)		0.233
γ-GTP (IU/L)	117	42	(11–545)		57	43	(11–431)		60	37	(12–545)		0.413
ALP (IU/L)	117	222	(24–462)		57	205	(24–340)		60	226	(48–462)		0.001
Cr (mg/dL)	117	0.82	±	0.20	57	0.80	±	0.20	60	0.80	±	0.20	0.594
eGFR (mL/min/1.73m^2^)	117	69.6	±	16.5	57	71.3	±	17.3	60	67.9	±	15.7	0.260
FLI	117	69.3	(8.6–97.7)		57	69.7	(8.9–95.8)		60	64.8	(8.6–97.7)		0.479

The data are presented as n (%) or medians (Min-Max) or means ± SDs. *P* values are for comparison between the two groups. BMI, body mass index; TG, triglyceride; HDL-C, high-density lipoprotein cholesterol; LDL-C, low-density lipoprotein cholesterol; IRI, immunoreactive insulin; HOMA-IR, homeostasis model assessment insulin resistance; AST, aspartate aminotransferase; ALT, alanine aminotransferase; γ-GTP, gamma-glutamyl transpeptidase; ALP, alkaline phosphatase; eGFR, estimated glomerular filtration rate; FLI, fatty liver index.

### 3.2 Changes in FLI from baseline to week 16

**Figure 1:**
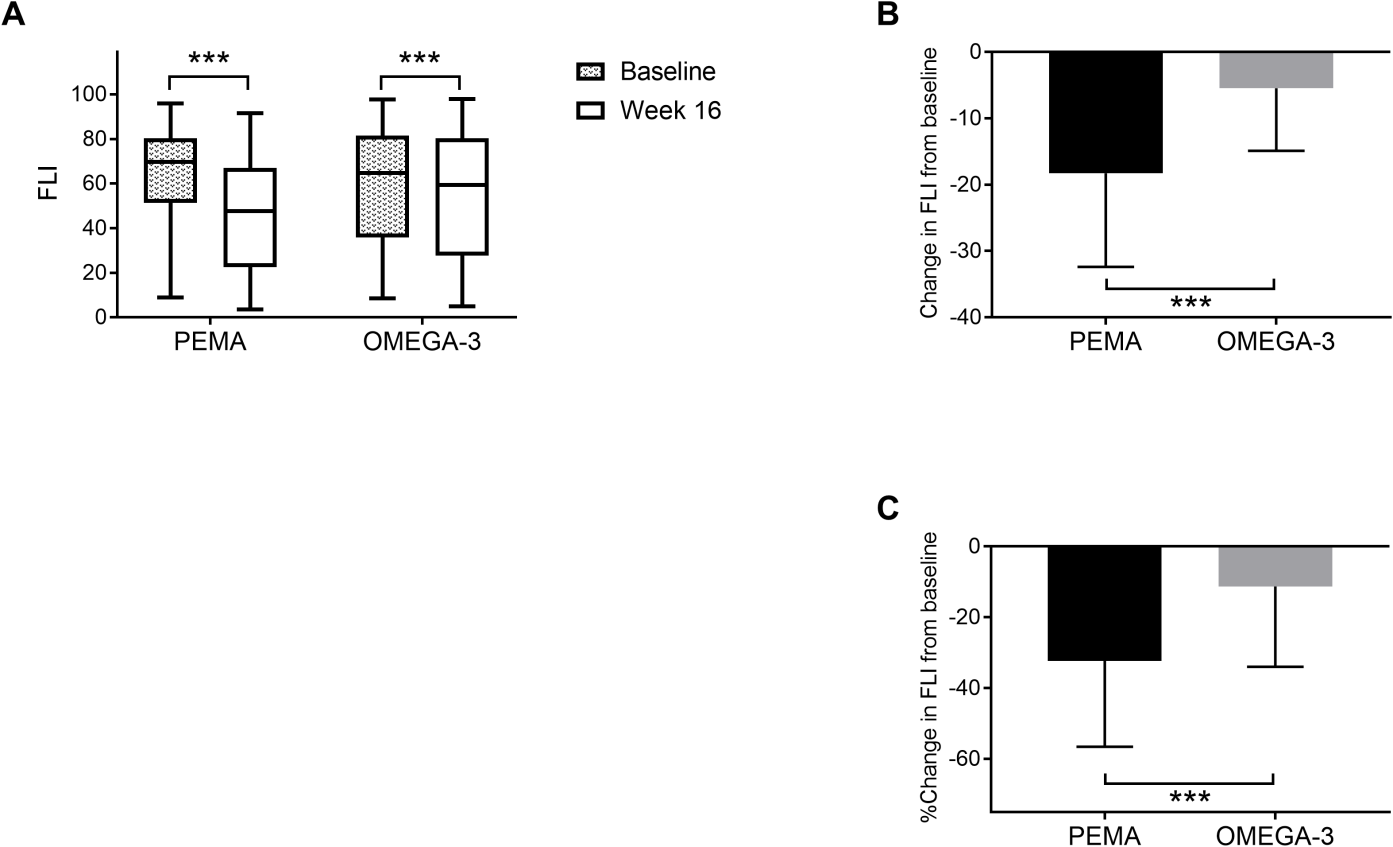
Change in FLI from baseline to week 16. **(A)** FLI at baseline and week 16. **(B)** Change in FLI. **(C)** Percentage change in FLI. The data are presented as medians (Min-Max) **(A)** or means ± SDs **(B, C)**. The boxes indicate the interquartile ranges **(A)**. ***P < 0.001 for comparisons between baseline and week 16 **(A)**. ***P < 0.001 for comparisons between PEMA and OMEGA-3 **(B, C)**. FLI, fatty liver index; PEMA, pemafibrate treatment group; OMEGA-3, omega-3 fatty acid ethyl treatment group.

From baseline to week 16, FLI was significantly decreased in both the PEMA group (69.7 [IQR 51.2, 80.4] to 47.6 [IQR 22.6, 67.1], *P* < 0.001) and the OMEGA-3 group (64.8 [IQR 35.8, 81.5] to 59.5 [IQR 27.6, 80.3], *P* < 0.001) (**Figure 1A**). The change in FLI in the PEMA group and that in the OMEGA-3 group were -18.3 ± 14.1 and -5.5 ± 9.4 (*P* < 0.001), respectively (**Figure 1B**). The percentage change in FLI in the PEMA group and that in the OMEGA-3 group were -32.3 ± 24.3% and -11.3 ± 22.7 (*P* < 0.001), respectively (**Figure 1C**).

**Figure 2:**
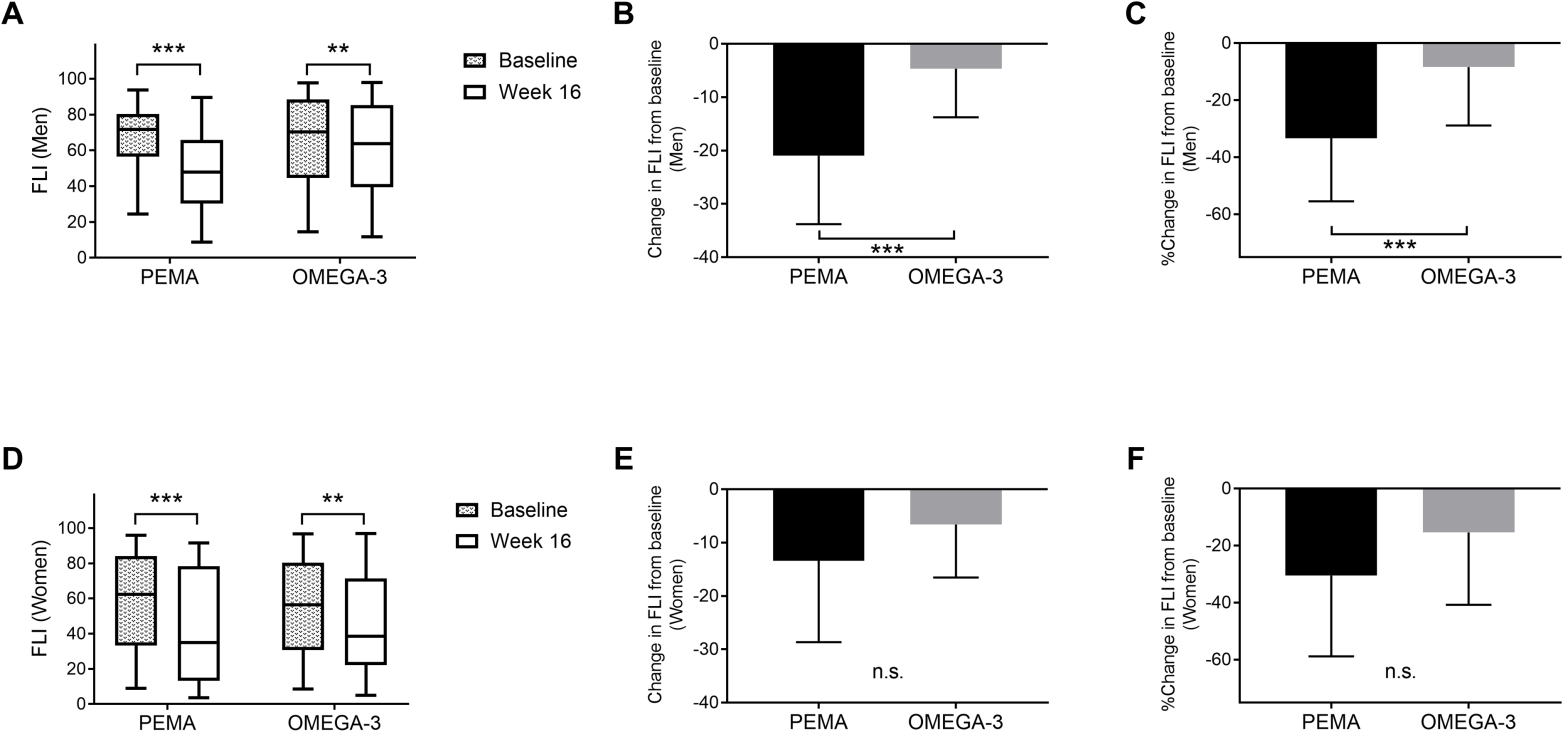
Change in FLI from baseline to week 16 divided by sex. **(A)** FLI at baseline and week 16 in men. **(B)** Change in FLI in men. **(C)** Percentage change in FLI in men. **(D)** FLI at baseline and week 16 in women. E: Change in FLI in women. F: Percentage change in FLI in women. The data are presented as medians (Min-Max) **(A, D)** or means ± SDs **(B, C, E, F)**. The boxes indicate the interquartile ranges **(A, D)**. **P < 0.01, ***P < 0.001 for comparisons between baseline and week 16 **(A, D)**. ***P < 0.001 for comparisons between PEMA and OMEGA-3 **(B, C)**. FLI, fatty liver index; PEMA, pemafibrate treatment group; OMEGA-3, omega-3 fatty acid ethyl treatment group; n.s., not significant.

**Figure 2** shows changes in FLI from baseline to week 16 in both treatment groups divided by sex. In men, FLI was significantly decreased in both the PEMA group (71.7 [IQR 56.4, 80.4] to 47.9 [IQR 30.3, 65.9], *P* < 0.001) and the OMEGA-3 group (70.2 [IQR 44.6, 88.6] to 63.7 [IQR 39.4, 85.4], *P* = 0.005) (**Figure 2A**). The change in FLI in the PEMA group and that in the OMEGA-3 group were -20.9 ± 12.8 and -4.6 ± 9.1 (*P* < 0.001), respectively (**Figure 2B**). The percentage change in FLI in the PEMA group and that in the OMEGA-3 group were -33.3% ± 22.1 and -8.4% ± 20.4 (*P* < 0.001), respectively (**Figure 2C**).

In women, similar to the results in men, FLI was significantly decreased from baseline to week 16 in both the PEMA group (62.4 [IQR 33.3, 84.1] to 34.8 [IQR 13.2, 78.3], *P* < 0.001) and the OMEGA-3 group (56.4 [IQR 30.7, 80.3] to 38.6 [IQR 22.2, 71.5], *P* = 0.004) (**Figure 2D**). However, in women, there was no significant difference in the change in FLI or the percentage change in FLI between the treatment groups (**Figures 2E, F**).

### 3.3 The prevalence of MASLD

**Figure 3:**
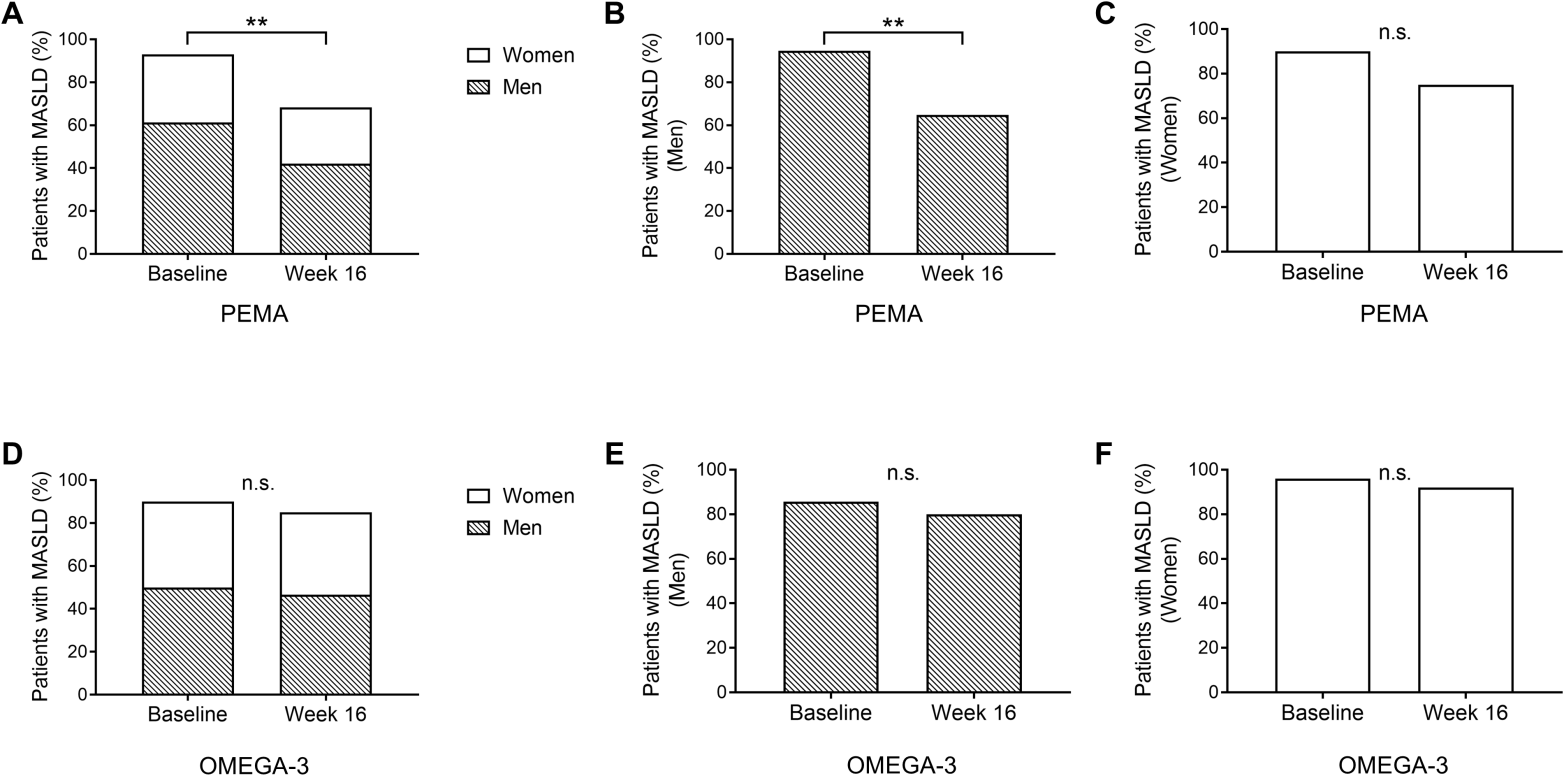
The prevalence of MASLD. **(A)** The proportion of patients with MASLD at baseline and week 16 in PEMA. **(B)** The proportion of male patients with MASLD at baseline and week 16 in PEMA. **(C)** The proportion of female patients with MASLD at baseline and week 16 in PEMA. **(D)** The proportion of patients with MASLD at baseline and week 16 in OMEGA-3. **(E)** The proportion of male patients with MASLD at baseline and week 16 in OMEGA-3. **(F)** The proportion of female patients with MASLD at baseline and week 16 in OMEGA-3. The data and bars are presented as percentages. **P < 0.01 for comparisons between baseline and week 16 **(A, B)**. MASLD: metabolic dysfunction-associated steatotic liver disease; PEMA, pemafibrate treatment group; OMEGA-3, omega-3 fatty acid ethyl treatment group; n.s., not significant.

In the PEMA group, the proportion of MASLD was significantly decreased from baseline to week 16 (93.0% to 68.4%, *P* = 0.002) (**Figure 3A**). When the patients were divided by sex, the proportion of MASLD was significantly decreased from baseline to week 16 in men (94.6% to 64.9%, *P* = 0.003) but not in women (90.0% to 75.0%, *P* = 0.408) (**Figures 3B, C**). On the other hand, in the OMEGA-3 group, there was no significant difference in the proportion of MASLD between the points of baseline (90.0%) and week 16 (85.0%) (*P* = 0.582) (**Figure 3D**). Even when the patients were divided by sex, there was no significant difference in proportion of MASLD between the points of baseline and week 16 (**Figures 3E, F**).

## 4 Discussion

To the best of our knowledge, this study for the first time showed direct comparison of the effects of two TG-lowering agents on FLI in patients with dyslipidemia receiving statin treatment. We revealed that both pemafibrate and omega-3 fatty acid ethyl significantly decreased FLI from baseline to week 16 and that the decrease in FLI was significantly greater in patients treated with pemafibrate than in patients treated with omega-3 fatty acid ethyl. In addition to previous findings in the PROUD48 study showing that pemafibrate was superior to omega-3 fatty acid ethyl in lowering effect of ALT, γ-GTP, and ALP levels, we newly demonstrated that pemafibrate was also superior to omega-3 fatty acid ethyl in the lowering effect of FLI, a surrogate marker for detection of MASLD, in this sub-analysis study of the PROUD48 study. In addition, a significant reduction in the prevalence of MASLD was found in patients treated with pemafibrate but not in those treated with omega-3 fatty acid ethyl. Taken together, the findings obtained in the present study further highlight the possibility that pemafibrate is a useful agent for not only hypertriglyceridemia but also NAFLD/MASLD as a potential ASCVD risk.

A recent review Jump et al. (22) summarized various clinical findings including randomized trials regarding the effects of omega-3 fatty acid ethyl on NAFLD/NASH assessed by lipids, liver function, surrogate markers, and imaging findings such as ultrasound and magnetic resonance imaging (MRI) (23–29). Some studies verified that omega-3 fatty acid ethyl improved histological findings in the liver in patients with NAFLD/NASH (30, 31). On the other hand, although pemafibrate is a new TG-lowering agent, single-arm preliminary studies conducted in Japan showed that pemafibrate decreased levels of ALT, γ-GTP and ALP and improved surrogate markers for liver fibrosis and cirrhosis (32–36). In addition, a recent double-blind, placebo-controlled, randomized phase 2 trial conducted in Japanese patients with NAFLD showed that pemafibrate significantly improved MRI-based liver stiffness and reduced levels of ALT and surrogate markers for liver fibrosis compared with placebo, although treatment with pemafibrate failed to reduce MRI-based liver fat content (18). Thus, both two agents are potentially useful for the treatment of NAFLD/MASLD.

In the present study, pemafibrate was superior to omega-3 fatty acid ethyl in the lowering effect of FLI. Additionally, a reduction in the prevalence of MASLD was observed in only patients treated with pemafibrate. It has recently reported that pemafibrate was superior to omega-3 fatty acid ethyl in not only lowering ALT, the primary endpoint, but also improving other liver enzymes, lipid profiles, and hepatic fibrosis biomarkers in the PORTRAIT study, which prospectively compared the effects of pemafibrate and omega-3 fatty acid ethyl on liver function in patients with hypertriglyceridemia complicated by MASLD (37). Although backgrounds of participants, including the prevalence of MASLD, liver function, proportion of statin treatment, and the dose and treatment period, were different between the PROUD48 study and PORTRAIT study, these two studies provided an important evidence that pemafibrate is a promising drug for improving MASLD compared to omega-3 fatty acid ethyl.

Intrahepatic lipid content is normally regulated by a balance between lipid uptake and disposal in the liver (38). In NAFLD/MASLD, pathways of hepatic lipid metabolism are dysregulated as follows: 1) increased hepatic lipid uptake, 2) increased *de novo* lipogenesis, 3) decreased fatty acid oxidation, and 4) increased VLDL production, resulting in hepatic lipid accumulation (38). It has been reported that both pemafibrate and omega-3 fatty acid ethyl increase fatty acid oxidation in the liver, thereby reducing the fatty acid pool that is the source of hepatic lipid accumulation (39, 40). Treatment with omega-3 fatty acid ethyl has also been reported to reduce fatty acid pool by suppressing *de novo* lipogenesis in the liver (40). These effects of the two agents on hepatic lipid metabolism should be effective against NAFLD/MASLD.

In NAFLD/MASLD, both an increase in dietary fatty acids due to overnutrition and an increase in fatty acids due to enhanced lipolysis in peripheral adipose tissue are associated with insulin resistance, leading to increased hepatic lipid uptake (38). The latter has been reported to be the major source of lipid accumulation in the liver in patients with NAFLD (41). In the PROUD48 study, the median levels of HOMA-IR at baseline in the PEMA group and the OMEGA-3 group were 2.34 and 2.68, respectively, suggesting that the participants in both groups were mildly insulin resistant (5). Although there was no significant difference in change in HOMA-IR level from baseline to week 16 between the PEMA group and the OMEGA-3 group, HOMA-IR level was significantly decreased by only the treatment with pemafibrate (5). The improvement in insulin resistance by pemafibrate may result in the suppression of lipolysis in adipose tissue, leading to reduction of the fatty acid pool in the liver and subsequent improvement of NAFLD/MASLD. In addition, the favorable impact of pemafibrate on insulin resistance may be also involved in the reduction of dietary fatty acids via its effect on lipoprotein lipase (38, 42). Furthermore, we previously showed that pemafibrate was superior to omega-3 fatty acid ethyl in lowering effect of fasting apoB-48 as a surrogate marker that reflects postprandial hypertriglyceridemia (5). The greater effect of pemafibrate in lowering postprandial TG-rich lipoproteins may lead to a decrease in the absolute amount of TG derived from the gut, which accounts for most dietary lipids, and a reduction in its mobilization to the liver as dietary fatty acids (43).

In the present study, the median levels of FLI at baseline in the PEMA group and the OMEGA-3 group were 69.7 and 64.8, respectively. Since the levels of FLI in both groups were ≥ 60, which met the originally reported cutoff level for diagnosis of NAFLD in Italian subjects (14), it would be acceptable to consider most of the participants in the present study to have NAFLD/MASLD. It has been reported that the optimal FLI for predicting NAFLD was lower in Japanese subjects than in Italian subjects and that there was a sex difference in the cutoff level of FLI (≥ 35 for men and ≥ 16 for women) (17). Therefore, in the present study, we adopted the cutoff values for Japanese subjects in the detection of MASLD. Interestingly, the present study showed that the beneficial effects of pemafibrate compared with omega-3 fatty acid ethyl on MASLD were predominant in men. A previous study using a rodent model showed that hepatic PPARα expression was predominant in male rats compared with female rats (44). This report may help us understand why pemafibrate may be more beneficial than omega-3 fatty acid ethyl for NAFLD/MASLD. However, a recent retrospective study using Japanese patients with NAFLD showed that the beneficial effects of pemafibrate on NAFLD were predominant in women (45). This issue of possible sex difference in the efficacies of pemafibrate remains controversial and requires further investigation.

This study has several limitations. First, although FLI has been shown to correspond to histological findings of NAFLD/MASLD, it is nothing more than a surrogate marker for NAFLD. It is possible that changes in FLI as well as the prevalence of MASLD observed in this study merely reflected changes in parameters that constitute the equation of FLI. In fact, in the PROUD48 study, pemafibrate significantly reduced levels of TG and γ-GTP compared to omega-3 fatty acid ethyl (5). To compare the true effect of these two agents on MASLD, prospective comparative trial should be conducted preferably with histological outcomes rather than surrogate markers or biomarkers. Second, although FLI is an established biomarker for the detection and prediction of NAFLD/MASLD (12, 15–17), the change in FLI has not been validated as a clinical indicator reflecting alterations in the pathophysiology of NAFLD/MASLD. Therefore, certain caution is required in interpreting the results observed in this study. Third, there were concerns about the diagnostic process for MASLD. The diagnosis of MASLD requires the exclusion of other discernible causes for hepatic steatosis (10). Although we interviewed all participants and reviewed their medical records to confirm the existence of comorbid liver diseases, we were unable to perform liver imaging or testing for the presence of hepatitis viruses in all participants. Therefore, we could not exclude the possibility that some participants in this study had SLD associated with other etiologies. Fourth, since the present study was a sub-analysis of the PROUD48 study, the participants were patients with dyslipidemia. Therefore, some of the patients did not have diagnosis of MASLD. Fifth, approximately half of the participants had diabetes, and some of them were treated with glucose-lowering agents including sodium-glucose cotransporter-2 inhibitors and glucagon-like peptide-1 receptor agonists, which may affect hepatic steatosis. Sixth, as this study was an open-label trial, there were concerns about potential bias and its impact on the results. Finally, the lack of placebo or combination treatment group was also a limitation in the present study.

In conclusion, pemafibrate is superior to omega-3 fatty acid ethyl in lowering effects of FLI and the prevalence of MASLD estimated by FLI in patients with dyslipidemia receiving statin treatment. Pemafibrate would be a beneficial TG-lowering agent for reducing risk of ASCVD as well as MASLD compared to omega-3 fatty acid ethyl.

## Data Availability

The raw data supporting the conclusions of this article will be made available by the authors, without undue reservation.
